# Rapid High-Sensitivity Analysis of Methane Clumped Isotopes (Δ^13^CH_3_D and Δ^12^CH_2_D_2_) Using Mid-Infrared Laser Spectroscopy

**DOI:** 10.1021/acs.analchem.4c05406

**Published:** 2025-01-08

**Authors:** Naizhong Zhang, Ivan Prokhorov, Nico Kueter, Gang Li, Béla Tuzson, Paul M. Magyar, Volker Ebert, Malavika Sivan, Mayuko Nakagawa, Alexis Gilbert, Yuichiro Ueno, Naohiro Yoshida, Thomas Röckmann, Stefano M. Bernasconi, Lukas Emmenegger, Joachim Mohn

**Affiliations:** † Laboratory for Air Pollution/Environmental Technology, Empa, 8600 Dübendorf, Switzerland; ‡ Department of Earth and Planetary Science, ETH Zurich, 8092 Zürich, Switzerland; § Department General and Inorganic Chemistry, PTB, 38116 Braunschweig, Germany; ∥ Department Analytical Chemistry of the Gas Phase, PTB, 38116 Braunschweig, Germany; ⊥ Institute for Marine and Atmospheric Research Utrecht, Utrecht University, Utrecht 3584CC, The Netherlands; # Department of Earth and Planetary Sciences, Institute of Science Tokyo, 152-8551 Tokyo, Japan; ∇ Earth-Life Science Institute, Institute of Science Tokyo, 152-8550 Tokyo, Japan; ○ National Institute of Information and Communications Technology, 184-8795 Tokyo, Japan

## Abstract

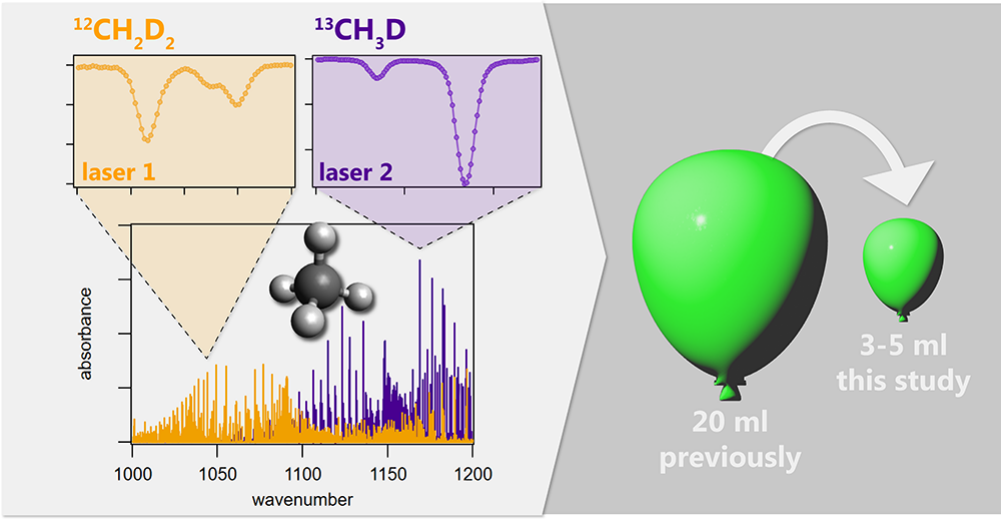

Mid-infrared laser absorption spectroscopy enables rapid and nondestructive analysis of methane clumped isotopes. However, current analytical methods require a sample size of 20 mL STP (0.82 mmol) of pure CH_4_ gas, which significantly limits its application to natural samples. To enhance the performance of spectroscopic measurement of methane clumped isotopes, we established a laser spectroscopic platform with newly selected spectral windows for clumped isotope analysis: 1076.97 cm^–1^ for ^12^CH_2_D_2_ and 1163.47 cm^–1^ for ^13^CH_3_D, and a custom-built gas inlet system. These spectral windows were identified through an extensive spectral survey on newly recorded high-resolution Fourier transform infrared (FTIR) spectra across the wavelength range of 870–3220 cm^–1^, thereby addressing gaps for ^12^CH_2_D_2_ in existing spectral databases. In addition, we implemented several key technological advances, which result in superior control and performance of sample injection and analysis. We demonstrate that for small samples ranging from 3 to 10 mL (0.12–0.41 mmol) of CH_4_ gas, a measurement precision comparable to high-resolution isotope ratio mass spectrometry for Δ^12^CH_2_D_2_ (∼1.5‰) can be achieved through 3 to 8 repetitive measurements using a recycle-refilling system within a few hours. Samples larger than 10 mL can be quantified in under 20 min. At the same time, for Δ^13^CH_3_D analysis a repeatability of 0.05‰, superior to mass spectrometry, was realized. These advancements in reducing sample size and shortening analysis time significantly improve the practicality of the spectroscopic technique for determining the clumped isotope signatures of natural methane samples, particularly for applications involving low CH_4_ concentrations or requiring consecutive analyses, which are feasible in conjunction with an automated preconcentration system.

Methane (CH_4_) is an important energy resource,^1^ a crucial component in biogeochemical cycles^2^ and a potent greenhouse gas (GHG), with high mitigation potential toward achieving climate agreements.^3^ Understanding its sources and sinks is essential for predicting its role in both current and past global carbon cycles. This knowledge can also aid in the search for potential energy reservoirs and in exploring the possibility of evidence for life on other planets, such as Mars.^4^ Determining the stable isotope ratios of carbon (^13^C/^12^C, referred to as δ^13^C–CH_4_) and hydrogen (D/H, referred to as δD–CH_4_),^5^ combined with measurements of other hydrocarbons like ethane and propane, have provided extensive insights into CH_4_ production pathways.^6^ However, due to the complexity of CH_4_ origins, these signatures are often not conclusive, and novel approaches are needed to enhance our understanding of natural CH_4_ cycling.

Since 2004, the concept of analyzing multiply substituted isotopologues (also known as “clumped” isotopes) has gained attention.^7^ For example, the ^13^C–^18^O bond in carbonate is used as a palaeothermometry tool.^8^ In the case of methane, two doubly substituted species—^13^CH_3_D and ^12^CH_2_D_2_—are expected to provide complementary information about either formation or postgeneration temperature under equilibrium conditions, or about formation or consumption pathways/processes under disequilibrium conditions.^9−12^ Over the past decade, techniques for quantifying methane clumped isotopes have advanced, using high-resolution isotope ratio mass spectrometry (e.g., 253 Ultra HR-IRMS^9,13−15^ and Panorama HR-IRMS^16−19^) and quantum cascade laser absorption spectrometer (TILDAS or QCLAS).^20,21^ Due to the low abundance of ^12^CH_2_D_2_ (0.14 ppm), precise analysis by HR-IRMS typically requires more than 12 h, making the entire analytical process for a single methane sample approximately 20 h long.^13,14^ In contrast, Gonzalez et al.^21^ demonstrated that the laser spectroscopic approach could reduce measurement time to around 4 h with improved precision. However, the relatively large sample size (∼20 mL STP) required for this procedure still limits its broader application, especially for atmospheric studies, where CH_4_ separation from around 10^4^ L of air would be needed.

To enhance the performance of laser spectroscopic analysis of methane clumped isotopes, particularly ^12^CH_2_D_2_, with respect to sample volume requirement and throughput, we established a laser spectroscopic platform by selecting optimized spectral windows for ^12^CH_2_D_2_ (1076.97 cm^–1^) and ^13^CH_3_D (1163.47 cm^–1^) and implementing a customized gas inlet system with highly accurate pressure and temperature control. Selection of spectral windows was based on newly recorded high-resolution FTIR spectra of ^13^CH_3_D (1000–3220 cm^–1^) and ^12^CH_2_D_2_ (870–3190 cm^–1^), made available for future use. The developed analytical platform offers analysis of δ^13^C–CH_4_, δD–CH_4_, Δ^13^CH_3_D and Δ^12^CH_2_D_2_ at performance compatible to HR-IRMS, but in a fraction of the time and with less than half the sample volume required for earlier QCLAS instruments.

## Experimental Section

### Notation for Bulk and Clumped Isotope Analysis

The bulk carbon and hydrogen stable isotopic ratios of CH_4_ are reported relative to international standards, Vienna Pee Dee Belemnite (VPDB) and Vienna Standard Mean Ocean Water (VSMOW), respectively in conventional delta notation, as follows
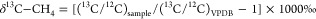
1
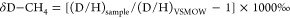
2

The clumped isotopic composition of CH_4_, denoted as Δ, is defined as the deviations in the abundances of mass-18 isotopologues relative to the stochastic distribution of each isotope
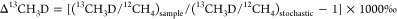
3
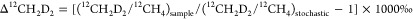
4

At equilibrium, the distribution of isotopes in doubly substituted CH_4_ isotopologues is governed by two isotope exchange reactions
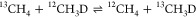
5
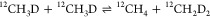
6The equilibrium constants for eqs 5 and 6 follow the principles of statistical thermodynamics. Therefore, the relationship between clumped isotopic abundances and temperature can be predicted using *ab initio* molecular calculations.^13,17,22,23^ In this study, we used the relationship reported by Young et al.^17^

### Investigation of Spectral Regions

While spectral data of more abundant CH_4_ isotopologues, such as ^12^CH_4_, ^13^CH_4_, ^12^CH_3_D and ^13^CH_3_D, are readily available in spectral databases like HITRAN^24^ or GEISA,^25^ data for ^12^CH_2_D_2_ are still missing. Gonzalez et al.^21^ selected spectral lines for ^12^CH_2_D_2_ based on model simulations, as no absorption cross sections were available.^26−28^

**Figure 1:**
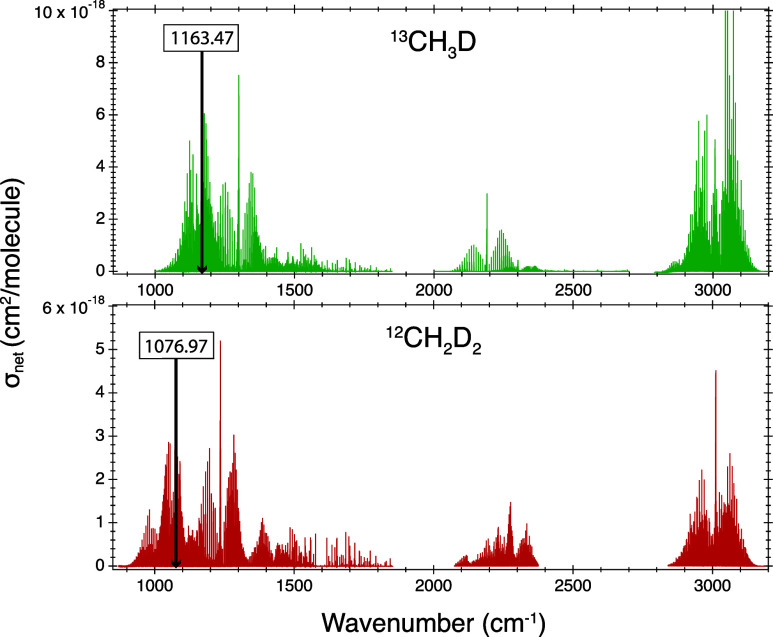
Net absorption cross sections of ^13^CH_3_D and ^12^CH_2_D_2_ isotopologues in mid-IR spectral region analyzed by high resolution FTIR spectroscopy. Vertical black lines indicate the spectral windows selected for clumped isotope analysis by QCLAS.

To guide the selection of ro-vibrational lines for both doubly substituted methane isotopologues, ^13^CH_3_D and ^12^CH_2_D_2_, high-resolution FTIR spectra were recorded over the wavelength range of 3.1 to 11.5 μm (870 to 3220 cm^–1^) at Physikalisch-Technische Bundesanstalt (PTB) in Braunschweig, Germany. For this, CH_4_ gases with high chemical purity (>98%) and high abundance in ^13^CH_3_D (99% ^13^C, 98% D, CDLM-9065-0, Cambridge Isotope Laboratories) or ^12^CH_2_D_2_ (98% D_2_, DLM-1343-0, Cambridge Isotope Laboratories) were analyzed. In addition, spectra of ultrahigh purity CH_4_ (99.9995%, Linde AG, Germany) at natural isotopic composition were analyzed as a reference. Spectra were recorded at resolutions between 0.0035 and 0.007 cm^–1^ over a range of pressures from 0.1 to 10 Torr at 296 K, using a White-type multipass cell with 0.85 m optical path length (Bruker Optics IFS 125HR). Figure 1 presents an overview graph of the recorded spectra given as net absorption cross sections. Further details are provided in Supporting Information S1.

By combining high-resolution FTIR spectra of ^13^CH_3_D and ^12^CH_2_D_2_ with HITRAN model simulations for more abundant isotopologues, a comprehensive data set was established and absorption lines were selected for clumped isotopic measurements (Table 1), as will be discussed and evaluated in the following sections.

**Table 1:** Spectroscopic Parameters of the Absorption Lines Selected for This Study^a^

species	ν (cm^–1^)	*S* (cm/molecule)	*E*″ (cm^–1^)	γ (cm^–1^)
laser 1				
^12^CH_3_D	1076.8447	1.416 × 10^–25^	313.1903	0.08
^12^CH_2_D_2_	1076.9698	1.575 × 10^–27^		
laser 2				
^13^CH_3_D	1163.4737	5.94 × 10^–26^	7.7536	0.086
^12^CH_4_	1163.4937	3.27 × 10^–25^	2055.917	0.068
^13^CH_4_	1163.6288	1.395 × 10^–25^	950.1988	0.067

a All parameters are taken from the HITRAN2020 database, except for those of ^12^CH_2_D_2_. ν: line position; S: spectral line intensity; E″: lower state energy; γ: pressure broadening parameter.

### Spectrometer

The spectrometer deployed in this study is a customized dual-laser trace gas monitor (QCLAS, Aerodyne Research Inc.). Continuous-wave (cw) quantum cascade lasers (QCL, Alpes Lasers, Switzerland) were installed (L1:1076.83–1077.06 cm^–1^ at 17.36 °C, L2:1163.45–1163.65 cm^–1^ at 6.91 °C) as mid-infrared light sources. Laser temperatures are maintained by thermoelectric control-loops at about 0.001 K precision levels. The analyzer uses an astigmatic Herriott multipass cell with 413 m optical path length (∼2.5 L of volume, including dead volume). A heated capacitance manometer (Baratron AA02A, MKS) monitors the pressure in the optical cell. Temperature stability of the spectrometer is achieved by a two-stage procedure: the interior temperature of the spectrometer is maintained with a recirculating water chiller (Oasis, Solid State Cooling), while the entire instrument is enclosed in a custom-made plexiglass thermal shield (120 × 70 × 70 cm^3^), stabilized with a high-power Peltier-element assembly (Deltron Lairs AA-200), driven by a high-precision PID controller (Meerstetter, Switzerland). Temperature stabilization of about 0.1 K is achieved in the enclosure, despite temperature fluctuations in the laboratory environment of 2.5 K, while the temperature stability of the multipass cell and optical module is at the level of 2 mK over time scales of days.

### Gas Inlet System

**Figure 2:**
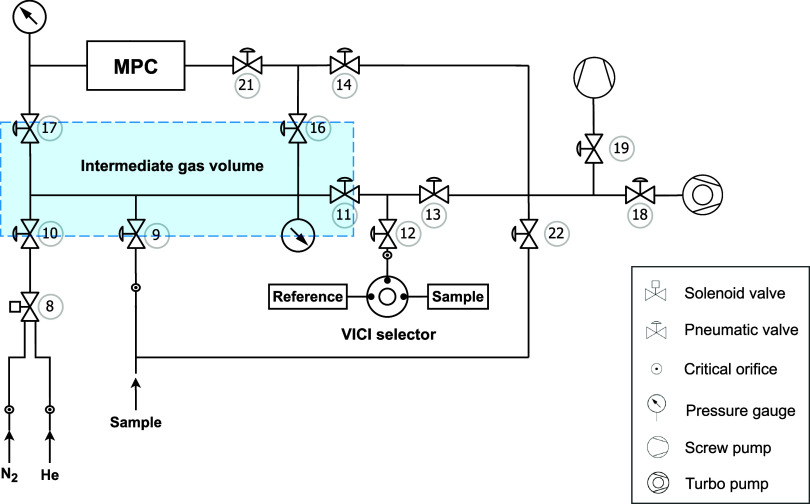
Schematic drawing of the custom-built inlet system for reference and sample injection/withdrawal into the spectrometer multipass cell (MPC). First, the gas fills the intermediate gas volume (light blue shaded area) via either valve 9 or the VICI selector valve. The gas flow into the intermediate volume is restricted by critical orifices and the filling process is terminated once the set pressure is reached. Valves No. 17 and 21 control the respective injection and extraction of the analyte gas into the MPC. Evacuation is accomplished with a screw pump and a turbomolecular pump operated sequentially.

Gases are introduced to the spectrometer with a custom-built fully automated gas inlet system (Figure 2). It consists of four normally closed bellow-sealed pneumatically actuated valves (SS-48K-1C, Swagelok) with 0.25 in. fittings to deliver high-purity (99.9999%) N_2_, sample, or reference gas to the intermediate expansion volume (∼50 mL), and eight valves of the same type with 0.5 in. VCR-gasket-sealed fitting (SS-8BK-VCR-1C, Swagelok) for gas handling, including injection to or extraction from the spectrometer cell. A screw vacuum pump (PDV 500, Ebara Corporation, Japan) is used for evacuating larger amounts of gas from the cell and inlet system, down to 0.1 Torr, and a turbo-molecular pump backed up by a diaphragm pump (HiCube 80 Eco, Pfeiffer Vacuum, Germany) for evacuation down to 0.1 mTorr. Reference gases are introduced into the system through a 16-port dead-end selector valve (Valco Vici AG). Every second input port of the valve is blinded and used as a parking position, while reference gases are plumbed to intermediate positions.

Under standard operation each reference/sample pair was preceded by a background spectrum measurement of N_2_ collected at 1.5 times the target reference/sample pressure to compensate for the different refractive index between gases.^21^ Next, the laboratory working reference gas (EP6) was analyzed at the target pressure (200 s), followed by the measurement of the sample gas at the same pressure (200 s). Optimal spectral averaging times and maximum time gaps between sample and reference gas measurements were chosen based on the results of Allan-Werle deviation measurements. During background, reference or sample preparation, gas was expanded into the intermediate volume until a target pressure is reached (15.5 Torr per mL CH_4_), controlled using a 0–1000 Torr manometer (Baratron AA02A, MKS) and a critical orifice. The repeatability of pressure readings for the intermediate volume is better than 0.5 Torr (1 σ standard deviation). For CH_4_ samples of 10 mL or larger, the pressure of the intermediate volume (>155 Torr) can be adjusted to better than 0.4% difference between reference and sample, ensuring measurement precision and accuracy are unaffected. For smaller samples, e.g., 3 mL of CH_4_ (pressure in the intermediate volume of 50 Torr), pressure differences between repeated analyses can reach up to 3.5%. At a spectrometer cell pressure of 1 Torr, pressure differences of 3.5% between reference/sample gas affect δ^13^C–CH_4_, δD–CH_4_, Δ^13^CH_3_D and Δ^12^CH_2_D_2_ by 0.12, 0.00, 0.12, and 2.55‰, respectively, which are within or comparable to overall instrumental analytical error. However, to enhance precision and accuracy, and particularly to avoid biases arising from varying sample amounts, a pressure correction has been applied to all measurements in this study (see S2.1 in Supporting Information for details).

### Equilibrated Gas Preparations

To equilibrate methane samples for instrumental calibration, we constructed a compact heated setup (Figure S5). In brief, 1.0–4.0 g of aluminum oxide (γ-Al_2_O_3_, 1/4″ pellets, Thermo Fisher Scientific) was placed inside a 120 mm long, electro-polished stainless-steel tube (o.d. 1/2 in.), sealed at both ends with 2 μm spring-retained frit filters (Swagelok) and high-temperature bellows-sealed valves (SS-8UW, Swagelok). The assembly was housed inside a brass jacket and heated using a ceramic wire heater (Wisag, Switzerland). The temperature of the reaction vessel was stabilized with a PID controller to better than ±1 K. The temperature gradient across the length of the reactor chamber was less than 1 K. For practical purposes, such as facilitating catalyst exchange, this equilibrium system was later modified by baking it inside an oven. Prior to loading the sample, the catalyst was activated under vacuum conditions (<1 mTorr) at 567 °C for 24 h.^29^ Around 3750 Torr (∼5 bar) pure methane gas was then introduced into the reaction chamber, sealed and equilibrated at a target temperature ranging from 70 to 300 °C. After equilibration, the methane sample was cryogenically extracted into a custom stainless-steel cold trap (∼10 mL, no absorbent, Swagelok) immersed in liquid nitrogen. We experimentally confirmed that this methane collection step does not introduce any detectable isotopic alteration beyond the analytical error (Table S5).

## Results and Discussion

### Selection of Spectral Windows and Analytical Advances

Using the high-resolution FTIR spectra of ^13^CH_3_D and ^12^CH_2_D_2_ obtained in this study, we were able to identify highly adequate spectral windows for clumped isotope analysis. The selection of spectral line positions for each clumped isotopologue aims to meet the following criteria: rotational lines should have high absorption cross section or line strength for maximal sensitivity; the selected ^13^CH_3_D and ^12^CH_2_D_2_ lines must be neighbored by equally strong lines of the major isotopologues (^12^CH_4_, ^13^CH_4_, ^12^CH_3_D) for referencing; and they must be sufficiently separated to minimize spectral interferences.

**Figure 3:**
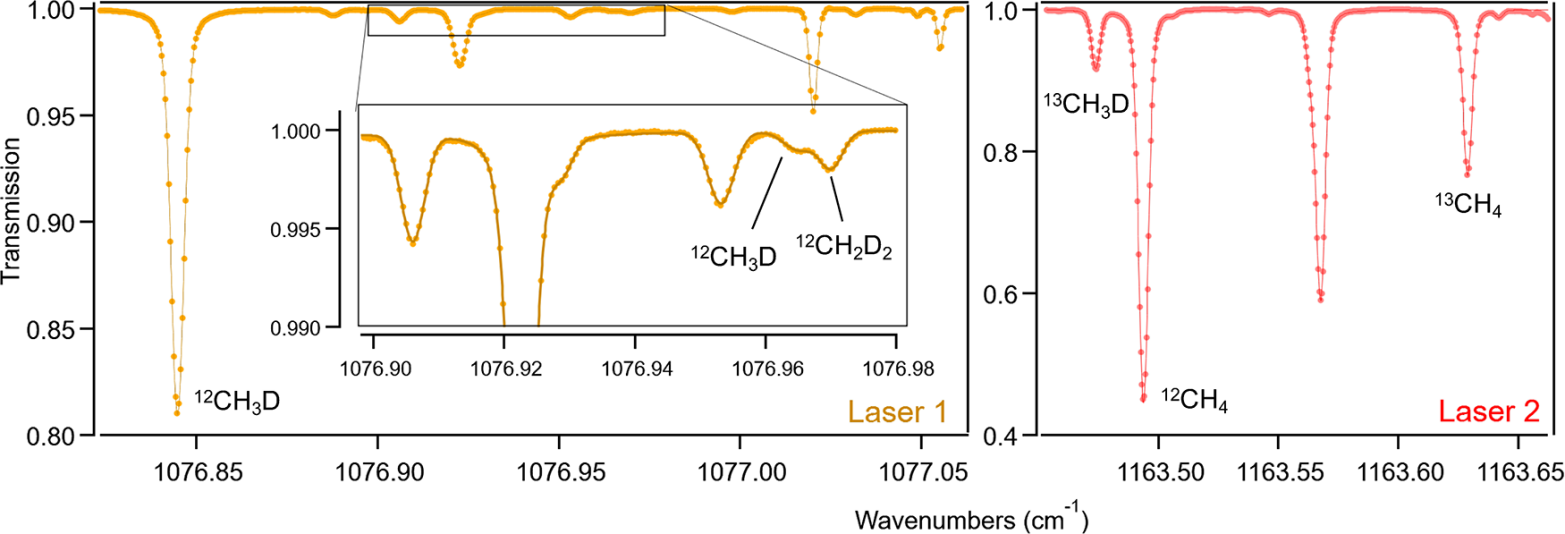
Measured (point) and fitted (line) transmission spectrum in the spectral range covered by laser 1 and laser 2 at 7.5 Torr cell pressure of pure methane.

Figure 1 indicates that for ^12^CH_2_D_2_, the maximum net absorption cross sections in the 900 to 1600 cm^–1^ range occur around 1235 cm^–1^ (5.2 × 10^–18^ cm^2^/molecule). However, this spectral range is characterized by very strong absorption of the main CH_4_ isotopologues, making it unsuitable for selective ^12^CH_2_D_2_ analysis. Several ro-vibrational lines of ^12^CH_2_D_2_ offer absorption cross sections in the range of 2.0–3.0 × 10^–18^ cm^2^/molecule. We selected a ^12^CH_2_D_2_ line at 1076.97 cm^–1^, with an absorption cross section of 2.9 × 10^–18^ cm^2^/molecule (spectral line intensity of 1.575 × 10^–27^ cm/molecule, Table 1), and analyzed it along with a ^12^CH_3_D line at 1076.84 cm^–1^. The spectral line intensity of the selected ^12^CH_2_D_2_ line is about 50% higher than the ^12^CH_2_D_2_ doublet selected by Gonzalez et al. (1090.39 cm^–1^, 1.068 × 10^–27^ and 6.79 × 10^–28^ cm/molecule).^21^ The higher spectral line intensity, along with the improved fitting of an individual line compared to a doublet, enhances sensitivity and precision, as demonstrated below. Spectral interference occurs from a ^12^CH_3_D line at 1076.96 cm^–1^ (Figure 3), which was not anticipated based on the available HITRAN data, but it is still much less pronounced compared to the earlier study, which was affected by the tail of a ^12^CH_4_ peak.^21^ Additionally, our newly published absorption cross sections offer opportunities to explore alternative spectral windows for ^12^CH_2_D_2_ analysis in the 1000–1600 cm^–1^ range at similar sensitivity, in the 2100–2400 cm^–1^ range at ≥50% reduced sensitivity, but with potentially higher selectivity. The 2850–3150 cm^–1^ range also displays high absorption cross sections, but does not offer separated ^12^CH_2_D_2_ lines.

For ^13^CH_3_D analysis, a line at 1163.47 cm^–1^ was selected, with a line intensity of 5.94 × 10^–26^ cm/molecule, similar to the line chosen by Gonzalez et al. (1200.26 cm^–1^).^21^ Due to the much higher natural abundance of ^13^CH_3_D compared to ^12^CH_2_D_2_, spectral interferences are less critical, and relevant effects can be predicted using spectral simulations. Along with ^13^CH_3_D, we analyzed ^12^CH_4_ and ^13^CH_4_ at 1163.49 and 1163.63 cm^–1^, respectively. The absorption spectra recorded at 7.5 Torr are shown in Figure 3.

In addition to spectroscopic advances discussed above, our analytical platform incorporates several technological advances, particularly with respect to the inlet system and laser spectrometer. These improvements enable increased sensitivity, facilitating analysis at reduced CH_4_ amounts. A key feature of the inlet system is the intermediate gas volume (Figure 2), where sample and reference gases are conditioned for temperature and pressure before injection into the spectrometer’s multipass cell. With high-precision pressure measurements and temperature control, we reduced pressure variations in the intermediate volume to ±0.5 Torr, which corresponds to ±0.01 Torr in the spectrometer sample cell. This is an order of magnitude better than the previously described instrument, which used a bellows-controlled sample inlet system that maintained cell pressure differences within ±0.1 Torr.^21^ Especially at lower sample amounts, i.e., low cell pressures, pressure differences between reference and sample gas measurements become the key limiting factor for precision and accuracy (see discussions in S2 for details) and complicated analysis at lower cell pressures/sample amounts in earlier systems.

Second, by implementing a two-stage temperature control system, the inlet system is stabilized to ±0.1 K and the spectrometer cell to ±2 mK, minimizing variations in the injected sample amount and reducing spectroscopic artifacts. Despite these precautions, additional factors, such as the operation of a fume hood within the same room, were observed to affect the precision of clumped isotope analysis using QCLAS, particularly for Δ^12^CH_2_D_2_. Avoiding such environmental perturbations can significantly improve QCLAS performance (see S2.2 for details). Third, the dead volume of the spectrometer multipass cell was minimized: the total cell volume, including connecting lines, was reduced to 2.5 L, compared to the 2.8 L reported by Gonzalez et al.,^21^ which corresponds to a ∼10% reduction in CH_4_ amount without sacrificing performance.

In summary, we introduced a number of new key analytical features, particularly the selection of an alternative spectral window for Δ^12^CH_2_D_2_ analysis with stronger line strength, that support superior performance and improved sensitivity.

### Instrumental Precision

**Figure 4:**
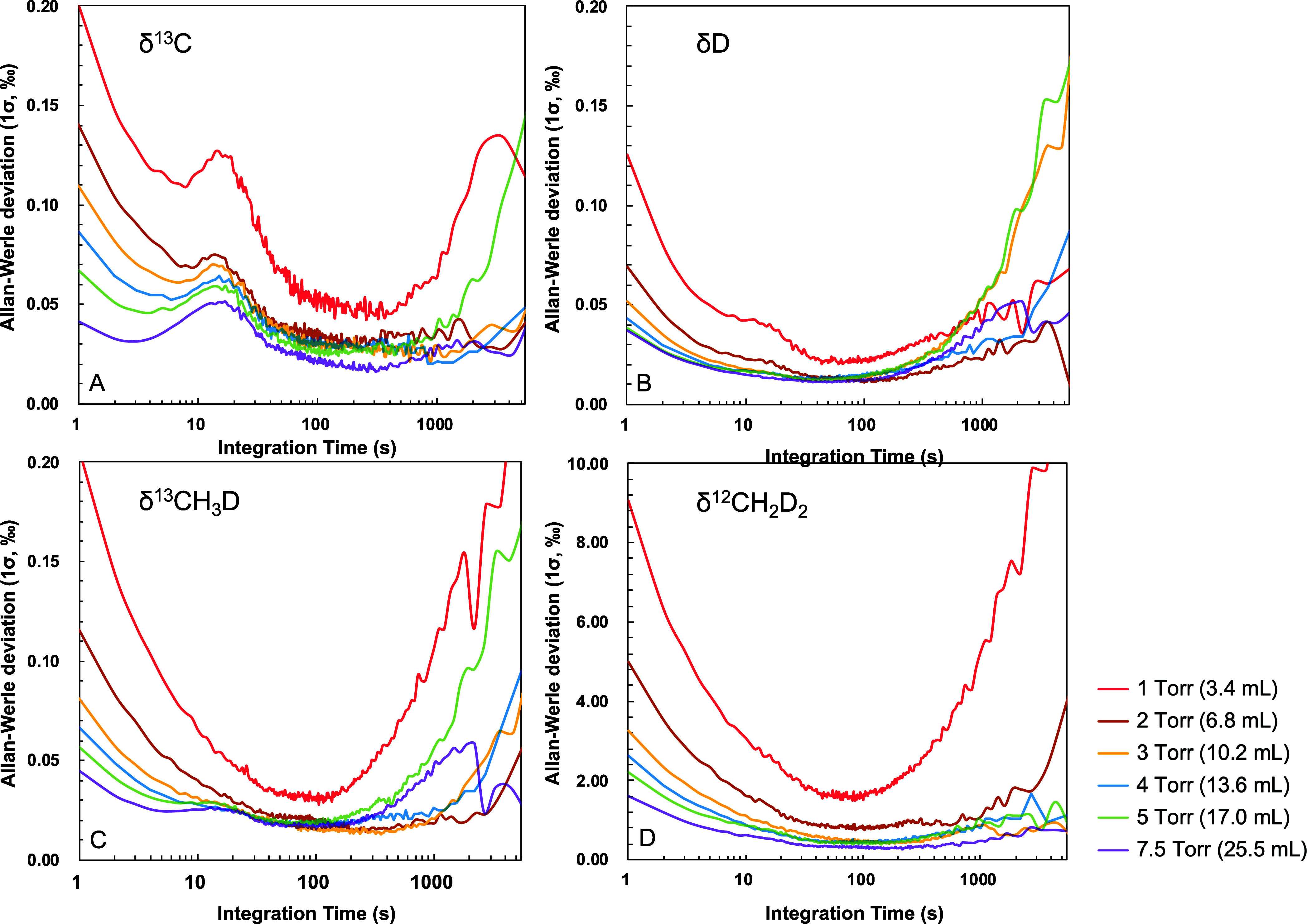
Allan-Werle deviation for individual isotope deltas at different cell pressures, i.e., methane sample amounts.

The measurement precision of the QCLAS was evaluated for sample amounts ranging from 3 to 25 mL of pure CH_4_ (0.12 to 1.03 mmol CH_4_), corresponding to pressures of 1 to 7.5 Torr in the spectrometer multipass cell. The CH_4_ amounts referenced here and throughout represent the methane filled in the multipass cell, including the inlet and outlet lines, without accounting for the ∼10% loss in the intermediate volume. The Allan-Werle variance technique^30^ was used for precision assessment. Sample measurements were preceded by background spectra correction using a N_2_-filled multipass cell, and then conducted over 2 h with a temporal resolution of one second. The calculated Allan deviations are presented in Figure 4 and Table S2, indicating white-noise limited behavior for approximately 10 s, followed by a steady improvement in precision up to an integration time of 100 to 150 s, at which best precision levels are achieved. For sample amounts larger than 10 mL CH_4_ (cell pressure above 3 Torr), the maximum precisions obtained were 0.03‰ for δ^13^C–CH_4_, 0.01‰ for δD–CH_4_, 0.02‰ for δ^13^CH_3_D, and 0.4‰ for δ^12^CH_2_D_2_, independent of sample amount. These values are comparable to those reported by Gonzalez et al.^21^ for 20 mL CH_4_. Since drift effects become significant after 1000 s spectral averaging, we implemented a standard procedure for a full measurement cycle consisting of background, reference and sample gas measurements with 200 s integration times each.

### Repeatability for Different CH_4_ Amounts

While the measurement precision of the laser spectrometer surpasses the precision reported for HR-IRMS, a more important factor with respect to applied performance is its repeatability for consecutive measurements of reference/sample pairs. By applying best practices in HR-IRMS, 1σ standard deviation values (1σ SD, external precision) of ∼0.3‰ for Δ^13^CH_3_D and ∼1.5‰ for Δ^12^CH_2_D_2_ can be achieved with a sample size of 3–5 mL per measurement.^13,14,16^ These values were used as a reference for our study. So far, spectroscopic measurements of Δ^12^CH_2_D_2_ have required much larger sample volumes, approximately 20 mL CH_4_, which complicates sample purification using standard gas chromatography columns and precludes its application for low methane concentration samples, such as atmospheric gas. Therefore, reducing the sample size for clumped isotope analysis using laser spectroscopy is crucial for its breakthrough in this field of research, taking advantage of its inherent capability for fast and nondestructive analysis.

**Figure 5:**
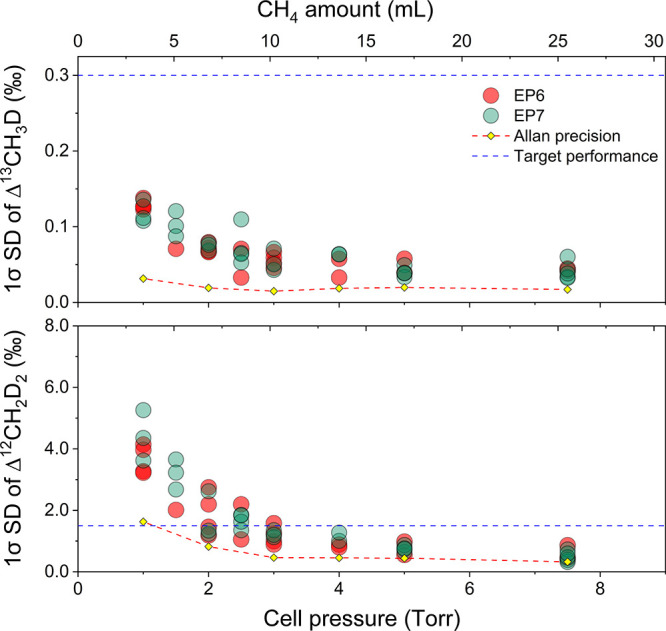
1σ standard deviation for repeated sample analysis applying different sample amounts (*n* = 20). Either EP6 or EP7 was used as the sample gas, while EP6 was used as reference for all analyses. The blue dashed line indicates the target performance, i.e., the 1σ SD (external precision) achieved by HR-IRMS (∼0.3‰ for Δ^13^CH_3_D and ∼1.5‰ for Δ^12^CH_2_D_2_). The red dashed line, along with the yellow diamonds, represents the Allan-Werle precision shown for comparison.

Figure 5 displays 1σ SD of Δ^13^CH_3_D and Δ^12^CH_2_D_2_ for repeated (*n* = 20) analyses of background-reference-sample pairs but using different amounts of CH_4_ reference and sample gas. In accordance with Allan-Werle deviation measurements, the CH_4_ amount ranged from 3 to 25 mL (0.12 to 1.03 mmol). Each full analysis was completed within 17 min.

For both Δ^13^CH_3_D and Δ^12^CH_2_D_2_ analyses, the repeatability (1σ SD) improved with increasing the amount of gas, but the improvement became negligible beyond ∼10 mL STP. Sample volume requirements for Δ^13^CH_3_D are much more relaxed with repeatability levels of ∼0.15‰ for 3 mL CH_4_ and ∼0.05‰ at 10 mL CH_4_ or higher, which is significantly better than the typical performance reported for HR-IRMS (∼0.3‰). The effect of sample amount on performance is more apparent for Δ^12^CH_2_D_2_, where the target repeatability (<1.5‰) could be reached for sample sizes larger than 10 mL (equivalent to >3 Torr cell pressure). A single 17 min-long analysis cycle is sufficient to reach this level, compared to typically measurement times of HR-IRMS of around 20 h. However, for sample sizes smaller than 10 mL (below 3 Torr cell pressure), the 1σ SD increased to ∼2‰ (7 mL) and over 4‰ at 3 mL CH_4_. In such cases, a recycle-refilling system connected to the current QCLAS system, such as the one reported in Gonzalez et al.,^21^ might be applied for 3 to 8 consecutive measurement cycles to achieve satisfactory external precision for Δ^12^CH_2_D_2_ analysis. Analysis time for 8 consecutive measurement cycles of <4 h^21^ is still a significant advancement as compared to HR-IRMS techniques.

In summary, we confirmed that the CH_4_ sample size required for spectroscopic clumped isotope analysis, to reach performance targets of 0.3‰ (or even as good as 0.05‰) for Δ^13^CH_3_D and 1.5‰ for Δ^12^CH_2_D_2_ can be reduced to 3–7 mL CH_4_, which is comparable to the sample size required for HR-IRMS analysis. Overall, an uncertainty of 0.05‰ in Δ^13^CH_3_D corresponds to an uncertainty of ±2 °C at an apparent temperature of 25 °C and of about ±5 °C at 200 °C, while an uncertainty of 1.5‰ in Δ^12^CH_2_D_2_ translates into an uncertainty of ±10 °C at 25 °C and ±50 °C at 200 °C, respectively.

### Heated Gas Calibration

Similar to HR-IRMS, the Δ^13^CH_3_D and Δ^12^CH_2_D_2_ values obtained through spectroscopic analysis are measured and calculated relative to a working reference gas (referred to as EP6). To determine the “true” clumped isotope signatures of samples, the clumped isotope values of the working reference gas must be quantified using equilibrated CH_4_ gases. These equilibrated gases can be prepared by heating a CH_4_ sample in the presence of a catalyst like Ni or γ-Al_2_O_3_ in a temperature-controlled and sealed system.^13,29^ In this study, we generated a series of equilibrated methane samples at 70, 150, 200 and 300 °C with varying bulk isotopic compositions, using the catalytic system described previously (Figure S6). Our observations indicate that the clumped isotope values reach equilibrium after 10 min at 300 °C and 45 min at 220 °C, while the bulk isotope values of the equilibrated CH_4_ gases remained consistent throughout the equilibration process (Figure S7). Consistency of δ^13^C–CH_4_ and δD–CH_4_, together with no significant change in absorption spectra, suggests no relevant CH_4_ decomposition over γ-Al_2_O_3_ under these conditions, eliminating the need for additional purification.

In principle, clumped isotope values should be independent of bulk isotope values. However, a significant nonlinearity effect, primarily influenced by δD–CH_4_ values, has been reported in previous spectroscopic analyses.^21^ To address this potential nonlinearity effects, we prepared a suite of in-house standard gases, including three commercially available pure methane gases (EP1, EP6, and EP7), and a ^12^CH_3_D-spiked sample gas (EP4). The δ^13^C–CH_4_ and δD–CH_4_ values of these reference gases are listed in Table 2. CH_4_ gases covering δD–CH_4_ values ranging from −204 to −40‰ facilitate to characterize dependencies of Δ^12^CH_2_D_2_ and Δ^13^CH_3_D on δD–CH_4_.

**Table 2:** Bulk and Clumped Isotope Values of Methane Gases Measured at Empa and HR-IRMS Laboratories (Tokyo Tech = Tokyo Institute of Technology, UU = Utrecht University)^a^

		*n*	δ^13^C^b^	error	δD^b^	error	Δ^13^CH_3_D	error	Δ^12^CH_2_D_2_	error
EP6^b^	Empa	36	–43.75		–188.72		3.58	0.02	8.90	0.23
	Tokyo Tech	3	–44.15	0.02	–196.56	0.08	3.65	0.10	7.19	1.57
	UU	1	–44.35	0.01	–185.80	0.16	3.11	0.33	7.28	1.50
EP1	Empa	20	–45.27	0.01	–203.89	0.01	4.10	0.01	8.63	0.19
	Tokyo Tech	3	–45.66	0.02	–211.30	0.01	3.92	0.13	9.43	0.94
	UU	1	–45.81	0.01	–201.30	0.05	3.94	0.25	9.15	1.30
EP7	Empa	20	–37.40	0.01	–163.24	0.01	2.55	0.01	3.76	0.07
	Tokyo Tech	3	–37.69	0.01	–171.44	0.03	2.31	0.11	3.38	1.63
	UU	1	–37.91	0.01	–160.70	0.10	2.78	0.25	3.09	1.30
EP6-HG300^c^	Empa	7	–43.76	0.07	–188.51	0.12	1.66	0.05	3.30	0.53
	Tokyo Tech	3	–44.02	0.21	–196.35	0.22	1.63	0.12	2.96	0.81
EP4	Empa	3	–46.21	0.00	–39.93	0.02	1.58	0.01	17.34	0.20

a The errors reported by Empa and Tokyo Tech represent the 1σ standard error, calculated based on the numbers of repetitions (external precision). In contrast, the errors reported by UU are calculated based on the number of reference-sample cycles within a single measurement (internal precision).

b δ^13^C–CH_4_ and δD–CH_4_ measurements at Empa are based on EP6, which has been previously analyzed at ETH Zurich against CH_4_ #1, 2, 3, 5, 7 reference gases provided by the Biogeochemical Laboratories at Indiana University.

c EP6 equilibrated at 300 °C.

**Figure 6:**
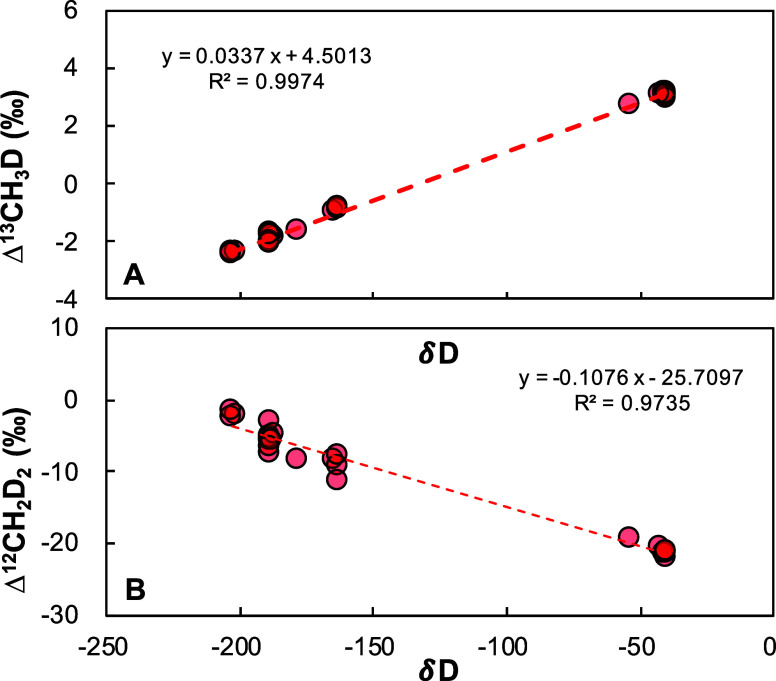
Apparent (A) Δ^13^CH_3_D and (B) Δ^12^CH_2_D_2_ values for CH_4_ gas with varying δD–CH_4_, equilibrated at 300 °C. EP6 was used as the reference for all measurements, while sample gases with different δD–CH_4_ were applied.

Figure 6 illustrates the correlations between apparent clumped isotope values (Δ^13^CH_3_D and Δ^12^CH_2_D_2_) and δD–CH_4_ for four CH_4_ samples equilibrated at 300 °C. At a cell pressure of 7.5 Torr, we observed a bias in Δ^13^CH_3_D of 0.034‰ and in Δ^12^CH_2_D_2_ of −0.108‰ for each 1‰ difference in δD–CH_4_ of the sample compared to the reference gas. This effect might be related to imperfect spectral fitting, in particular for the interfering ^12^CH_3_D line on ^12^CH_2_D_2_ and/or inaccuracies in baseline corrections. Relationships remain constant over time, unless major adjustments to the spectroscopic setup or spectral fitting are undertaken, which underlines the robustness of our analytical platform. Based on these correlations, the clumped isotope values of the reference gas (EP6) were determined to be 3.58 ± 0.02‰ for Δ^13^CH_3_D and 8.90 ± 0.23‰ for Δ^12^CH_2_D_2_ (Table 2).

### Interlaboratory Comparison

**Figure 7:**
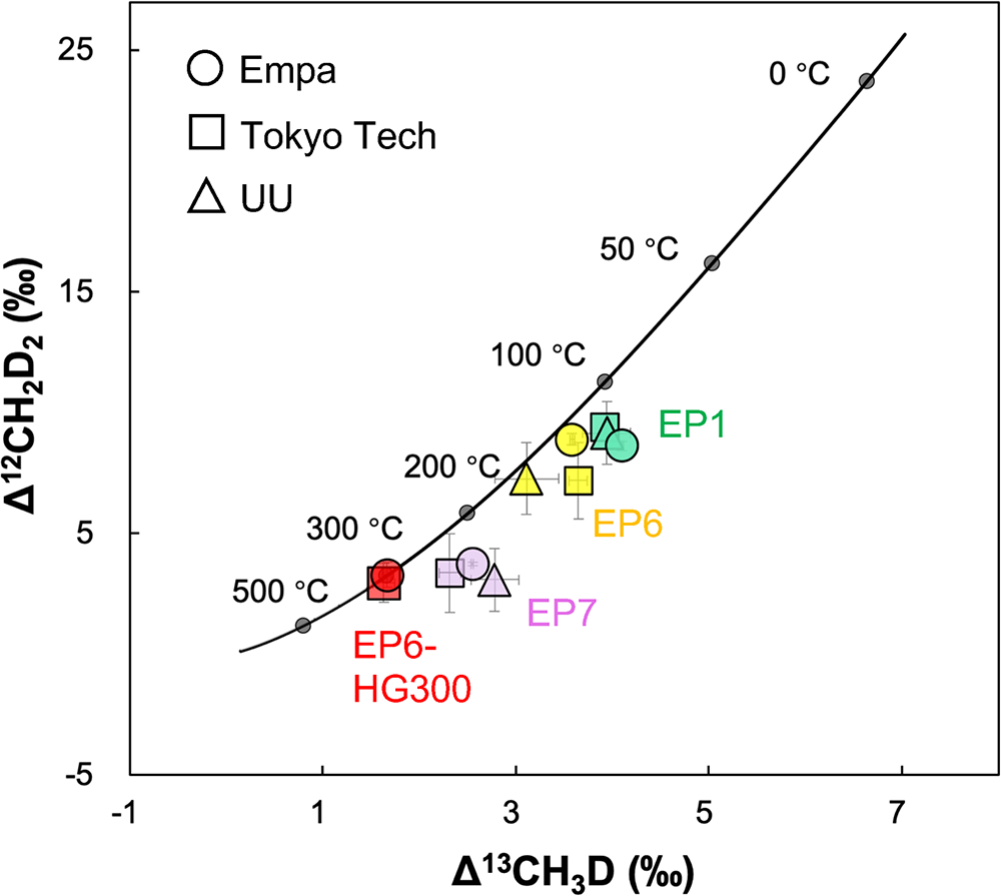
Comparison of methane clumped isotope analyses performed using different techniques (QCLAS vs HR-IRMS) across various laboratories. The black line indicates the theoretical values at thermodynamic equilibrium for different temperatures.^17^ Error bars represent 1σ standard error. Tokyo Tech: Tokyo Institute of Technology; UU: Utrecht University.

To assess the accuracy of clumped isotope analysis using QCLAS, our in-house standard methane gases (EP1, EP6, and EP7) and several equilibrated EP6 samples were analyzed using the 253 Ultra HR-IRMS (Thermo Fisher Scientific Inc.) at Tokyo Institute of Technology (Tokyo Tech, Japan; with the name changed to Institute of Science Tokyo from 1st, October, 2024) and Utrecht University (UU, The Netherlands). The results are shown in Table 2 and Figure 7. Overall, a systematic discrepancy in bulk isotope values was observed, with the δ^13^C–CH_4_ values measured by UU are approximately 0.5‰ higher than those measured at Empa, while the δD–CH_4_ values measured at Tokyo Tech are about 10‰ lower than those measured by UU and Empa. These discrepancies can be attributed to differences between the laboratories in referencing measurements to international isotope ratio scales.^31^

In contrast to bulk isotopes, both Δ^13^CH_3_D and Δ^12^CH_2_D_2_ values measured at Empa, Tokyo Tech, and UU show good agreement within the analytical uncertainty (within 2σ SE) reported by the laboratories, thereby validating the accuracy and compatibility of our spectroscopic technique for CH_4_ clumped isotope analysis with established methods that are more labor and cost intensive. Future efforts from the entire methane clumped isotope research community should focus on establishing a unified reference system to validate analyses across different analytical techniques and laboratories. Given the rapid analytical capabilities of laser-based techniques, it could facilitate the selection of suitable reference gases, whether through mixtures or spiking treatments, to support the efforts of minimizing interlaboratory biases.

## Conclusions

This study demonstrates a significant advancement in the spectroscopic measurement of methane clumped isotopes, particularly Δ^12^CH_2_D_2_, by reducing the required sample size while maintain repeatability and throughput. This was made possible through the refinement of spectral windows and optimization of a commercial QCLAS using a custom-built gas inlet system.

An extensive spectral survey for ^13^CH_3_D and ^12^CH_2_D_2_ was conducted using FTIR measurements across the spectral range of 870 to 3220 cm^–1^, addressing the existing gaps in available spectral databases for ^12^CH_2_D_2_. By combining these experimentally derived spectra with the HITRAN database, we identified new optimal spectral windows for clumped isotope analysis: 1076.97 cm^–1^ for CH_2_D_2_ and 1163.47 cm^–1^ for ^13^CH_3_D.

Testing the performance of the QCLAS system across various methane sample sizes revealed that at 10 mL CH_4_ (corresponding to 3 Torr cell pressure), the measurement precision and repeatability were comparable to, or even better than, those achieved with HR-IRMS. Specifically, the 1σ standard deviations were lower than 0.06‰ for both δ^13^C–CH_4_ and δD–CH_4_, 0.07‰ for Δ^13^CH_3_D, and 1.3‰ for Δ^12^CH_2_D_2_. This allows to substantially shorten the analysis time per sample, from about 20 h with HR-IRMS to about 17 min with QCLAS. For methane sample sizes below 10 mL, the precision for Δ^13^CH_3_D is still excellent, while for Δ^12^CH_2_D_2_ it dropped to around 2‰ at 2 Torr, and over 4‰ at 1 Torr. To achieve satisfactory repeatability for Δ^12^CH_2_D_2_ under these conditions, repeated analysis of a sample using a recycle-refilling system or consecutive analysis in conjunction with an automated preconcentration system such as CleanEx^32^ is feasible.

This advancement in QCLAS technology significantly reduces the sample size and time required for precise measurements of methane clumped isotopes. As a result, the spectroscopic technique is poised to become a more practical tool for analyzing samples from dissipated methane sources with low CH_4_ concentrations, such as atmospheric samples.

## Supporting Information Available

High-resolution FTIR spectra of ^13^CH_3_D and ^12^CH_2_D_2_; parameters controlling instrumental repeatability; additional figures and tables (PDF)
^13^CH_3_D net absorption cross sections (TXT)
^12^CH_2_D_2_ net absorption cross sections (TXT)
