# Correction to “Rapid High-Sensitivity Analysis of Methane Clumped Isotopes (Δ^13^CH_3_D and Δ^12^CH_2_D_2_) Using Mid-Infrared Laser Spectroscopy”

**DOI:** 10.1021/acs.analchem.5c02713

**Published:** 2025-05-15

**Authors:** Naizhong Zhang, Ivan Prokhorov, Nico Kueter, Gang Li, Béla Tuzson, Paul M. Magyar, Volker Ebert, Malavika Sivan, Mayuko Nakagawa, Alexis Gilbert, Yuichiro Ueno, Naohiro Yoshida, Thomas Röckmann, Stefano M. Bernasconi, Lukas Emmenegger, Joachim Mohn

The Acknowledgments in the original manuscript should be corrected to the following:

