# Supplementary material for: Rapid High-Sensitivity Analysis of Methane Clumped Isotopes (Δ13CH3D and Δ12CH2D2) Using Mid-Infrared Laser Spectroscopy
Source: Anal Chem. 2025 Jan 8;97(2):1291–9. doi: 10.1021/acs.analchem.4c05406 (PMC11755397; doi:10.1021/acs.analchem.4c05406)
Supplement: Supplementary file 1 — ac4c05406_si_001.pdf [file ac4c05406_si_001.pdf]

Supporting information

Rapid high-sensitivity analysis of methane clumped isotopes ($\Delta^{13}CH_3D$ and $\Delta^{12}CH_2D_2$) using mid-infrared laser spectroscopy

Naizhong Zhang*[,†,1], Ivan Prokhorov[1], Nico Kueter[2], Gang Li*[,3], Béla Tuzson[1], Paul M. Magyar[1], Volker Ebert[4], Malavika Sivan[5], Mayuko Nakagawa[6], Alexis Gilbert[6,7], Yuichiro Ueno[6,7], Naohiro Yoshida[7,8], Thomas Röckmann[5], Stefano Bernasconi[2], Lukas Emmenegger[1] and Joachim Mohn*[,1]

[1] *Laboratory for Air Pollution / Environmental Technology, Empa, 8600 Dübendorf, Switzerland*

[2] *Department of Earth and Planetary Science, ETH Zurich, 8092 Zürich, Switzerland*

[3] *Department General and Inorganic Chemistry, PTB, 38116 Braunschweig, Germany*

[4] *Department Analytical Chemistry of the Gas Phase, PTB, 38116 Braunschweig, Germany*

[5] *Institute for Marine and Atmospheric Research Utrecht (IMAU), Utrecht University, Utrecht 3584CC, The Netherlands*

[6] *Department of Earth and Planetary Sciences, Institute of Science Tokyo, 152-8551 Tokyo, Japan*

[7] *Earth-Life Science Institute, Institute of Science Tokyo, 152-8550 Tokyo, Japan*

[8] *National Institute of Information and Communications Technology, 184-8795 Tokyo, Japan*

Table of Contents

S1. High resolution FTIR spectra of $^{13}CH_3D$ and $^{12}CH_2D_2$; Figure S1 S2

S2. Parameters controlling instrumental repeatability; Figures S2 and S3 S4

S3. Supporting Figures S5-S7 S7

S4. Supporting Tables S1-S4 S9

Reference S12

S1 High resolution FTIR spectra of $^{13}CH_3D$ and $^{12}CH_2D_2$

This work makes available the first high-resolution rovibrational spectra of $^{12}CH_2D_2$ and improved spectra of $^{13}CH_3D$ to the community for further line-by-line analysis. It describes new high-resolution infrared absorption cross-sections of pure $^{12}CH_2D_2$ and $^{13}CH_3D$ over the spectral ranges of 870 – 3190 cm^{-1} and 1000 - 3220 cm^{-1}, respectively, derived from spectra recorded using a high-resolution Fourier transform spectrometer (Bruker Optics IFS 125HR). FTIR spectra were recorded at resolutions between 0.0035 cm^{-1} and 0.007 cm^{-1} over a range of pressures from 0.15 mbar to 13.5 mbar at 296 K using a 0.85 m multipass cell. In addition, natural CH_4 spectra were recorded subsequently using a 19.34 m White-type cell to assist line selection for laser spectroscopic analysis of methane clumped isotopes. Selection criteria were both good sensitivity and minimal spectral interference from major isotopologues of methane.

S1.1 Data availability prior to this study

While line parameters of $^{12}CH_4$, $^{13}CH_4$, $^{12}CH_3D$ and $^{13}CH_3D$ are available in HITRAN,[1] $^{12}CH_2D_2$ data are currently missing. Already in the 1970s, high-resolution FTIR spectra of $^{12}CH_2D_2$ were recorded and partial quantum assignments conducted.[2,3,4,5] Ulenikov et al.[6,7,8] revisited the $^{12}CH_2D_2$ spectra in the spectral range of 900 cm^{-1} to 6600 cm^{-1} by FTIR measurements at improved spectral resolution, but no absorption cross sections were published. In recent years, ab initio studies of $^{12}CH_2D_2$ were conducted, and provide the first predictions of rotationally resolved infrared spectra of $^{12}CH_2D_2$, but the root mean squared errors (RMSEs) of calculated frequencies were as high as 0.67 cm^{-1} [9] and 0.22 cm^{-1} [10], insufficient to assign rotational lines for laser spectroscopic analysis. The aim of the present study is to provide high-resolution spectra to support line selection in the mid-infrared spectral range for laser spectroscopic analysis of rare $^{12}CH_2D_2$ and $^{13}CH_3D$ along with more abundant $^{12}CH_4$, $^{13}CH_4$ and $^{12}CH_3D$ isotopologues.

S1.2 Materials and Methods

S1.2.1 FTIR infrastructure and measurements

The PTB central FTIR infrastructure has been applied, consisting of two Bruker Optics IFS 125HR FTIR spectrometers with peripheral equipment, described in more detail in Werwein et al..[11] For the present study two different optical multipass White-type cells have been adopted, one with a metal body (cell a: 0.8503 m, KBr wedged windows) and one with a glass body (cell b: 19.343 m, KBr wedged windows). The cell with metal body was temperature controlled with a refrigerated circulator (308, Huber Kältemaschinenbau, Germany) filled with ethanol, while the cell with glass body was temperature stabilized at 296 K using another refrigerated circulator (HE25, Julabo, Germany), filled with distilled water.

Prior to measurements, the cell and gas manifolds were filled with high purity nitrogen (99.9999% purity, Linde AG, Germany) to 1 bar and evacuated (HiCube Classic, Pfeiffer Vacuum, Germany). This procedure was repeated three times, before evacuation for one hour to remove residual nitrogen. Secondly, the gas cell was conditioned with 1 mbar of pure methane sample for 5 minutes before evacuation to 0.01 mbar. Lastly, pure methane sample gas was introduced into the cell to the desired pressures and measured with the FTIR spectrometer. The following CH_4 qualities were analyzed; $^{13}CH_3D$ (CDLM-9065-0, chemical purity: ≥98 %, isotopic purity: 99% ^{13}C, 98% D) and $^{12}CH_2D_2$ (DLM-1343-0, chemical purity: ≥98%, isotopic purity: 98 % D2) both purchased from Cambridge Isotope Laboratories (USA), pure CH_4 (99.9995%) acquired from Linde AG (Germany).

FTIR spectra were recorded at resolutions between 0.0035 and 0.007 cm^{-1}. In general, a mid-IR light source (Globar), a LN_2-cooled mercury cadmium telluride (MCT) detector and a potassium bromide (KBr) beam splitter were applied, while for measurement in the 3.3 μm region, a LN_2-cooled indium antimonide (InSb) detector was used instead for better response. Table S1 lists measurement conditions adopted for all spectra, i.e. sample pressure, temperature, targeted band, and spectral resolution together with the number of scans. Empty cell background interferograms were recorded at a lower resolution of 0.2 cm^{-1} before sample measurements. The lower resolution is justified since no spectra feature persists in the background measurements with an empty cell.

The sample pressure was measured using a Baratron capacitance manometer (full range 10 Torr, MKS Instruments, USA), which is regularly calibrated against a primary standard available at PTB with a relative uncertainty less than 0.2% (k=2). The cell temperature was measured by six platinum resistance thermometers (PT100) inserted into the pre-drilled hole on the cell body (2 cm in depth) for cell a. Considering the low sample gas pressure and the long waiting times before measurements

of around 10 minutes, the gas temperature is equal to the temperature of the thermal enclosure according to a recent computational fluid dynamics (CFD) simulation. Pressure and temperature readings were recorded at an interval of one minute using a customized LabVIEW program.

S1.2.1 Generation of absorption cross sections

By applying the Fourier-transform algorithm embedded in the Bruker OPUS7.5 software, measured interferograms were converted to sample singles in the frequency domain. To derive the net absorption cross section, first the transmission spectrum, T, is derived by dividing sample signal, I, with the background signal, I_0. Then transmission is converted to Naperian absorbance (A_e) via natural logarithm using Eq. S1. Finally, the net absorption cross-section (σ_{net}) is calculated using Eq. S2 by normalizing the Naperian absorbance with the actual optical path length (L), pressure (p) and temperature (T), and k_B being the Boltzmann constant (i.e. 1.3806488×10^{-23} J K^{-1}). Considering the low pressures used, the compression factor Z is taken as 1, i.e. ideal gas law applies.

$$A_e = -\ln(T) = -\ln\left(\frac{I}{I_0}\right) \tag{S1}$$

$$\sigma_{net} = A_e * \frac{k_B T}{p} * \frac{1}{L} \tag{S2}$$

S1.3 FTIR spectra of $^{13}CH_3D$ and $^{12}CH_2D_2$

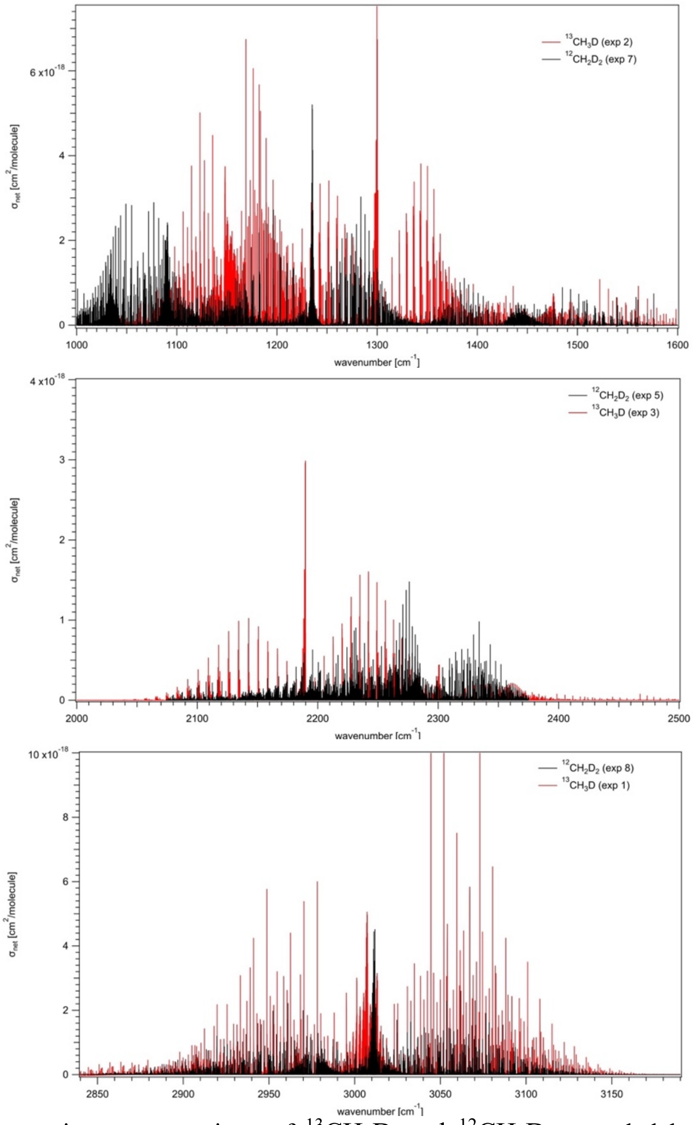

Figure S1. Net absorption cross-sections of $^{13}CH_3D$ and $^{12}CH_2D_2$ recorded by HR-FTIR at PTB Braunschweig.

S2. Parameters controlling instrumental repeatability

S2.1 Pressure dependence

The process of filling the sample into the spectrometer multipass cell at the desired pressure was achieved by first introducing the analyte gas into an intermediate volume using a manometrically controlled filling system. This procedure provided a repeatability better than 0.5 Torr (1σ SD) for the intermediate volume. At cell pressures above 3 Torr (intermediate volume pressure >155 Torr), pressure differences between the sample and reference gas were negligible, being less than 0.4%. However, at lower cell pressures, pressure differences became more significant. For instance, at a cell pressure of 1 Torr (intermediate volume pressure 53 Torr), the pressure difference between the sample and reference could reach as high as 3.5% (Figure S2).

The pressure imbalance introduced during the gas filling process, especially at low sample pressures, may impact the repeatability and accuracy of our clumped isotope analyses. To evaluate this effect, we examined the pressure dependence of each isotopic ratio across a range of cell pressures, from 1 Torr to 7.5 Torr, with the results presented in Table S3. Effects of pressure imbalances and goodness of fit (R^2) for $\delta^{13}C\text{-}CH_4$, $\delta D\text{-}CH_4$, and $\Delta^{13}CH_3D$, got more pronounced with higher cell pressures (Figure S3). However, the effect on $\Delta^{12}CH_2D_2$ was less consistent, likely due to the larger variability associated with $\Delta^{12}CH_2D_2$ analysis. Similar pressure effects have been reported by Gonzalez et al.[12]

To assess the potential analytical error arising from pressure imbalances during repeatability test, we compared the measured and corrected isotopic values at different cell pressures, with each pressure setting repeated 20 times (Table S4). The changes in isotopic values across all pressures remained within analytical error, and the improvement in repeatability (1σ SD) was relatively small, even at a cell pressure of 1 Torr. On the other hand, the pressure bias can become significant when the sample amount is near the minimum required to achieve the set cell pressure. For instance, a 0.5% difference between the sample and reference gas amounts at 7.5 Torr can lead to biases of approximately +0.09‰, -0.03‰, +0.16‰, and +0.24‰ in the measured $\delta^{13}C\text{-}CH_4$, $\delta D\text{-}CH_4$, $\Delta^{13}CH_3D$, and $\Delta^{12}CH_2D_2$ values, respectively. Given that the sample amount can vary case by case, applying pressure corrections is highly recommended to improve both precision and accuracy, particularly for $\Delta^{13}CH_3D$.

In summary, to maintain consistency, pressure corrections were applied to all measurements in this study.

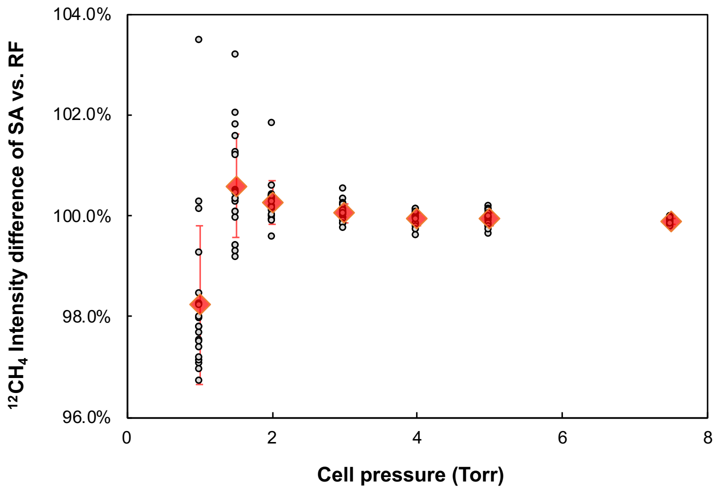

Figure S2. Pressure difference, represented as the intensity ratio of $^{12}CH_4$ between sample (SA) and reference (RF), for repeated injections (n = 20) at different cell pressures. Black circles represent individual SA – RF pairs, while red diamonds indicate the mean value for 20 repetitions.

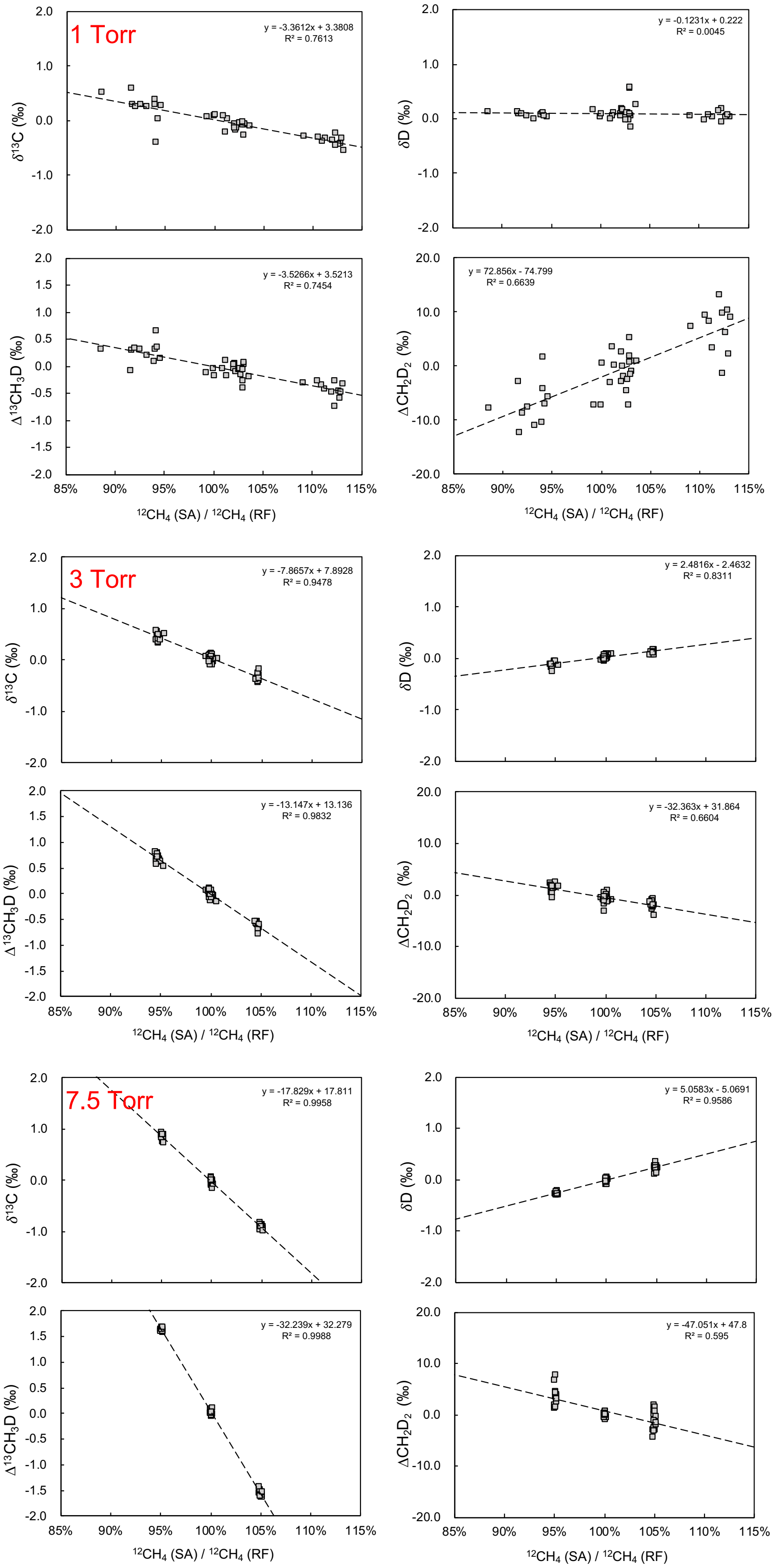

Figure S3. Pressure dependence of isotope deltas at 1, 3 and 7.5 Torr cell pressures for reference (RF) / sample (SA) pairs. The pressure differences on the x-axis are represented as the ratio of $^{12}CH_4$ concentrations for the sample and the reference gas.

S2.2 Other laboratory parameters

The precision and accuracy of isotopic analysis using a laser spectrometer are highly dependent on the stability of environmental parameters, such as temperature and pressure. To ensure optimal conditions, we designed and constructed a thermal enclosure capable of maintaining temperature variations within 0.1 K for the environment holding the spectrometer and the inlet system. This setup allows to stabilize the cell temperature within 2 mK, minimizing the impact of temperature fluctuations on both bulk and clumped isotope analyses during our experiments. Despite these precautions, we observed additional factors that may influence the precision of clumped isotope analysis using QCLAS, particularly for $\Delta^{12}CH_2D_2$. Among these factors, the operation of a fume hood in the experimental room emerged as a significant issue. The underlying cause of these interferences remains unclear, but it is likely related to pressure fluctuations induced by the fume hood, which may also affect the pressure inside the thermal enclosure and, consequently, deteriorate the performance of the laser measurement. During repeated measurements we noticed sporadic, extreme fluctuations in $\Delta^{12}CH_2D_2$ values, occurring exclusively during weekdays from 8:00 AM to 6:00 PM. As illustrated in Figure S4, we conducted 100 consecutive EP6 vs. EP6 measurements (zero test) at a cell pressure of 2 Torr (CH_4 of 6.8 mL STP). The fume hood was turned on after the 50th measurement. When the fume hood was off, the mean value for $\delta^{13}C$-CH_4, δD-CH_4, $\Delta^{13}CH_3D$, and $\Delta^{12}CH_2D_2$ were 0.03 ± 0.08‰, 0.01 ± 0.06‰, 0.02 ± 0.11‰, and -0.73 ± 2.24‰, respectively. However, with the fume hood on, these values changed to 0.02 ± 0.10‰, 0.06 ± 0.09‰, 0.02 ± 0.17‰, and -2.28 ± 6.63‰, respectively. Clearly, the repeatability (1σ SD) deteriorated significantly with the fume hood in operation. For $\Delta^{13}CH_3D$, the 1σ SD increased by 1.5 times compared to the expected value, while the 1σ SD for $\Delta^{12}CH_2D_2$ worsened by a factor of three.

To prevent such performance issues during QCLAS analysis, it is crucial to carefully monitor environmental parameters in the experimental room. Alternatively, conducting analyses in a dedicated room with minimal temperature / pressure fluctuations is highly recommended.

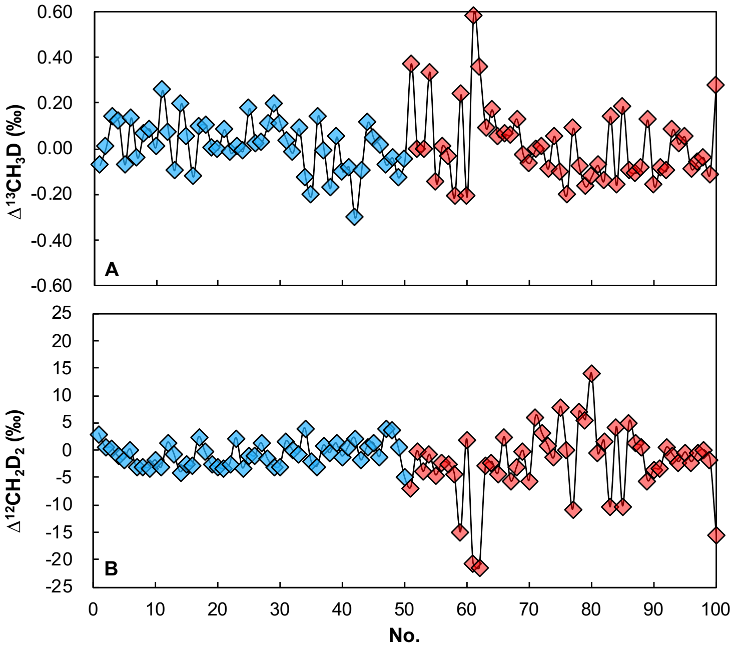

Figure S4. Variations in (A) $\Delta^{13}CH_3D$ and (B) $\Delta^{12}CH_2D_2$ during continuous EP6 vs EP6 (zero test) measurements, shown before (blue diamonds) and after (red diamonds) the fume hood was turned on (50th measurement cycle).

S3. Supporting Figures S5-S7

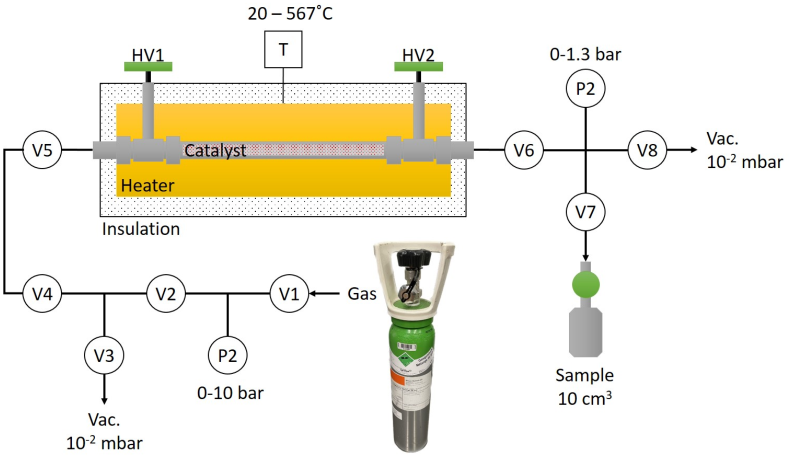

Figure S5. Schematic drawing of the setup applied for methane equilibration.

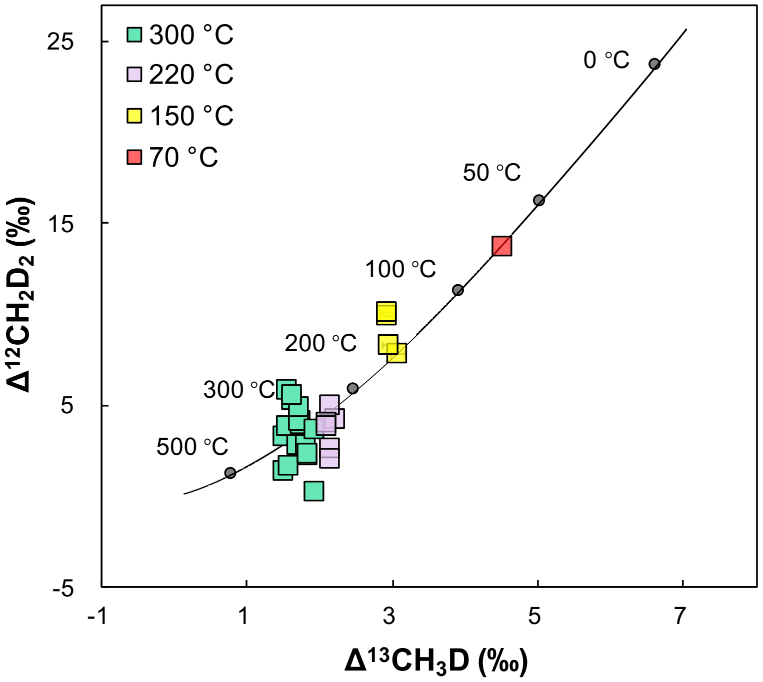

Figure S6. Experimental results of pure CH_4 gas equilibrated at different temperatures

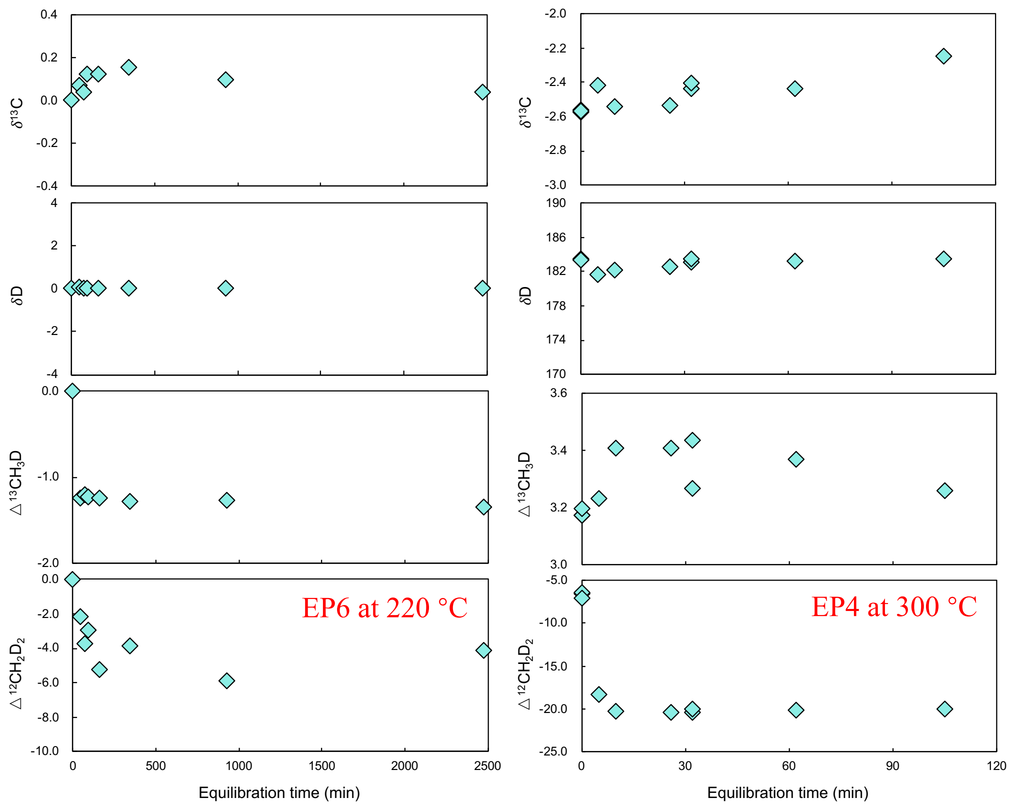

Figure S7. Changes in bulk and clumped isotope signatures of EP6 (vs. EP6) at 220 °C and EP4 (vs. EP6) at 300 °C with varying equilibration times. Equilibrium was reached after approximately 45 minutes at 220 °C and 10 minutes at 300 °C.

S4. Supporting Tables S1-S4

Table S1. Overview of the measurement conditions adopted for HR-FTIR analysis of $^{12}CH_2D_2$, $^{13}CH_3D$ and pure natural methane samples.

#	Molecule	Band (μm)	Cell	Temperature (K)	Calibrated Pressure (mbar)	Resolution (cm^{-1})	Aperture size (mm)	Total # of scans
1	$^{13}CH_3D$	3.11-3.58	a	296.0 ± 0.2	0.2695 ± 0.0030	0.0066	1.7	850
2	$^{13}CH_3D$	5.41-10.00	a	296.0 ± 0.2	0.1773 ± 0.0029	0.005	2	550
3	$^{13}CH_3D$	3.11-5.00	a	296.0 ± 0.2	0.7903 ± 0.0030	0.006	1.7	175
4	$^{13}CH_3D$	3.11-5.00	a	296.0 ± 0.2	13.46 ± 0.02	0.006	1.7	130
5	$^{12}CH_2D_2$	4.21-4.82	a	296.0 ± 0.2	0.6258 ± 0.0030	0.006	1.7	160
6	$^{12}CH_2D_2$	4.47-5.08	a	296.0 ± 0.2	13.44 ± 0.02	0.006	1.7	160
7	$^{12}CH_2D_2$	5.39-11.48	a	296.0 ± 0.2	0.1508 ± 0.0029	0.0035	1.7	360
8	$^{12}CH_2D_2$	3.13-3.52	a	296.0 ± 0.2	0.2309 ± 0.0030	0.007	1.7	120
9	Natural CH_4	2.99-3.55	b	296.1 ± 0.2	78.63 ± 0.04	0.01	2.5	1850
10	Natural CH_4	7.04-8.62	b	296.1 ± 0.2	78.19 ± 0.04	0.01	3.15	1600
11	Natural CH_4	8.26-10.53	b	296.1 ± 0.2	78.63 ± 0.04	0.01	3.15	2200
12	Natural CH_4	4.18-4.78	b	296.1 ± 0.2	99.75 ± 0.04	0.011	2.5	1600

cell a: 0.8503 ± 0.0020 m, KBr wedged windows
cell b: 19.343 ± 0.002 m, KBr wedged windows

Table S2. Allan-Werle precisions (1s, 100-150 s) observed for different methane amounts. Along with the CH_4 amount, pressure in the multipass cell (P_{cell}) and the intermediate volume (P_{inter}) are given. The CH_4 amount represents methane filled in the spectrometer multipass cell including inlet/outlet lines, while loss in the intermediate volume is not considered.

CH_4 amount	P_{cell}	P_{inter}	1s Allan deviation (‰)				Best Allan deviation (100-150 s, ‰)			
(mL STP)	(Torr)	(Torr)	$\delta^{13}C$	δD	$\delta^{13}CH_3D$	$\delta^{12}CH_2D_2$	$\delta^{13}C$	δD	$\delta^{13}CH_3D$	$\delta^{12}CH_2D_2$
3.4	1	53	0.20	0.13	0.21	9.06	0.05	0.02	0.03	1.63
6.8	2	105	0.14	0.07	0.12	4.98	0.03	0.01	0.02	0.82
10.2	3	158	0.11	0.05	0.08	3.27	0.03	0.01	0.02	0.46
13.6	4	210	0.09	0.04	0.07	2.62	0.03	0.01	0.02	0.45
17.0	5	263	0.07	0.04	0.06	2.19	0.02	0.01	0.02	0.44
25.5	7.5	395	0.04	0.04	0.04	1.62	0.02	0.01	0.02	0.33

Table S3. Observed correlations between isotope deltas and $^{12}CH_4$ concentration ratios for sample vs. reference gas, as a consequence of different sample amounts. Measurements were performed at different cell pressure (1-7.5 Torr).

	Slope	Intercept	R^2
**1 Torr**			
$\delta^{13}C$	-3.361	3.381	0.76
δD	-0.123	0.222	0.00
$\Delta^{13}CH_3D$	-3.527	3.521	0.75
$\Delta^{12}CH_2D_2$	72.856	-74.799	0.66
**1.5 Torr**			
$\delta^{13}C$	-3.570	3.584	0.84
δD	0.868	-0.827	0.56
$\Delta^{13}CH_3D$	-8.139	8.166	0.95

$\Delta^{12}CH_2D_2$	67.062	-68.444	0.64
**2 Torr**			
$\delta^{13}C$	-5.593	5.628	0.95
δD	0.654	-0.616	0.23
$\Delta^{13}CH_3D$	-9.034	8.999	0.97
$\Delta^{12}CH_2D_2$	53.740	-54.027	0.50
**3 Torr**			
$\delta^{13}C$	-7.866	7.893	0.95
δD	2.482	-2.463	0.83
$\Delta^{13}CH_3D$	-13.147	13.136	0.98
$\Delta^{12}CH_2D_2$	-32.363	31.864	0.66
**4 Torr**			
$\delta^{13}C$	-9.924	9.931	0.98
δD	4.777	-4.755	0.94
$\Delta^{13}CH_3D$	-18.841	18.836	0.99
$\Delta^{12}CH_2D_2$	-50.341	50.131	0.70
**5 Torr**			
$\delta^{13}C$	-11.919	11.927	0.98
δD	6.178	-6.161	0.96
$\Delta^{13}CH_3D$	-23.442	23.439	1.00
$\Delta^{12}CH_2D_2$	27.609	-26.843	0.47
**7.5 Torr**			
$\delta^{13}C$	-17.829	17.811	1.00
δD	5.058	-5.069	0.96
$\Delta^{13}CH_3D$	-32.239	32.279	1.00
$\Delta^{12}CH_2D_2$	-47.051	47.800	0.60

Table S4. Comparison of measured and corrected isotopic values of EP7 at different cell pressures (n = 20). All isotopic values are related to the reference gas (EP6).

P_{cell} (Torr)		$\delta^{13}C$-CH_4	1σ SD	δD-CH_4	1σ SD	$\Delta^{13}CH_3D$	1σ SD	$\Delta^{12}CH_2D_2$	1σ SD
1	Measured	6.71	0.08	31.45	0.07	-1.05	0.17	-7.63	3.73
	Corrected	6.75	0.10	31.35	0.07	-0.98	0.14	-7.07	3.62
2	Measured	6.64	0.09	31.34	0.04	-0.76	0.09	-8.98	1.39
	Corrected	6.62	0.08	31.30	0.04	-0.69	0.08	-8.89	1.34
3	Measured	6.74	0.06	31.41	0.04	-0.68	0.06	-7.78	1.17
	Corrected	6.71	0.05	31.39	0.03	-0.68	0.05	-7.32	1.20
7.5	Measured	6.66	0.04	31.43	0.04	-0.17	0.04	-6.68	0.60
	Corrected	6.67	0.04	31.45	0.04	-0.21	0.04	-7.43	0.60

Table S5. Comparison of measured isotope values (EP4 vs. EP6) between direct injection of CH_4 (Direct) and collection using a ~10 mL customized stainless-steel cold trap with liquid nitrogen (LQ N_2).

	n	$\delta^{13}C$	1σ SD	δD	1σ SD	$\Delta^{13}CH_3D$	1σ SD	$\Delta^{12}CH_2D_2$	1σ SD
Direct	5	-2.60	0.08	183.42	0.03	3.18	0.06	-6.81	0.97
LQ N_2	4	-2.69	0.16	183.41	0.05	3.25	0.22	-8.13	2.71

Reference

(1) Gordon, I. E.; Rothman, L. S.; Hargreaves, e. R.; Hashemi, R.; Karlovets, E. V.; Skinner, F.; Conway, E. K.; Hill, C.; Kochanov, R. V.; Tan, Y. The HITRAN2020 molecular spectroscopic database. *Journal of quantitative spectroscopy and radiative transfer* **2022**, *277*, 107949.

(2) Deroche, J.-C.; Guelachvili, G. High resolution infrared spectrum of CH2D2: The ν1 and ν6 fundamental bands near 3000 cm^{-1}. *Journal of Molecular Spectroscopy* **1975**, *56* (1), 76-87.

(3) Deroche, J.; Graner, G.; Cabana, A. High-resolution infrared spectrum of CH2D2: The ν3 fundamental band at 7 μm. *Journal of Molecular Spectroscopy* **1975**, *57* (3), 331-347.

(4) Deroche, J.; Pinson, P. High-resolution infrared spectrum of CH_2D_2: The ν9 fundamental band at 8 μm. *Journal of Molecular Spectroscopy* **1975**, *58* (2), 229-238.

(5) Deroche, J.; Graner, G.; Bendtsen, J.; Brodersen, S. The ν5 and ν3 Raman bands of CH2D2. *Journal of Molecular Spectroscopy* **1976**, *62* (1), 68-79.

(6) Ulenikov, O.; Bekhtereva, E.; Grebneva, S.; Hollenstein, H.; Quack, M. High resolution Fourier transform spectroscopy of CH_2D_2 in the region 2350–2650 cm^{-1}: the bands ν5 +ν7, 2ν9, ν3 + ν4, ν3 + ν7 and ν 5 + ν9. *Physical Chemistry Chemical Physics* **2005**, *7* (6), 1142-1150.

(7) Ulenikov, O. N.; Bekhtereva, E. S.; Grebneva, S. V.; Hollenstein, H.; Quack, M. High-resolution rovibrational analysis of vibrational states of A2 symmetry of the dideuterated methane CH_2D_2: The levels v_5 and $v_7 + v_9$. *Molecular Physics* **2006**, *104* (20-21), 3371-3386.

(8) Ulenikov, O. N.; Bekhtereva, E. S.; Albert, S.; Bauerecker, S.; Hollenstein, H.; Quack, M. High-resolution near infrared spactroscopy and vibrational dynamics of dideuteromethane (CH_2D_2). *Journal of Physical Chemistry A* **2009**, *113* (10), 2218-2231.

(9) Yu, H. G. Accurate quantum dynamics calculations of vibrational spectrum of dideuteromethane CH_2D_2. *Journal of Chemical Physics* **2015**, *142* (19).

(10) Rey, M.; Nikitin, A. V.; Tyuterev, V. G. First predictions of rotationally resolved infrared spectra of dideuteromethane ($^{12}CH_2D_2$) from potential energy and dipole moment surfaces. *The Journal of Physical Chemistry A* **2015**, *119* (20), 4763-4779.

(11) Werwein, V.; Brunzendorf, J.; Li, G.; Serdyukov, A.; Werhahn, O.; Ebert, V. High-resolution Fourier transform measurements of line strengths in the 00^02-00^00 main isotopologue band of nitrous oxide. *Applied Optics* **2017**, *56* (11), E99-E105.

(12) Gonzalez, Y.; Nelson, D. D.; Shorter, J. H.; McManus, J. B.; Dyroff, C.; Formolo, M.; Wang, D. T.; Western, C. M.; Ono, S. Precise Measurements of $^{12}CH_2D_2$ by Tunable Infrared Laser Direct Absorption Spectroscopy. *Anal Chem* **2019**, *91* (23), 14967-14974.
